# Molecular survey of *Cytauxzoon* spp. and *Hepatozoon* spp. in felids using a novel real-time PCR approach

**DOI:** 10.3389/fvets.2023.1113681

**Published:** 2023-06-12

**Authors:** Marika Grillini, Paola Beraldo, Antonio Frangipane di Regalbono, Giorgia Dotto, Cinzia Tessarin, Giovanni Franzo, Erica Marchiori, David Modrý, Giulia Simonato

**Affiliations:** ^1^Department of Animal Medicine, Production and Health, University of Padua, Padua, Italy; ^2^Department of Agricultural, Food, Environmental and Animal Sciences, University of Udine, Udine, Italy; ^3^Department of Veterinary Sciences, Faculty of Agrobiology, Food and Natural Resources/CINeZ, Czech University of Life Sciences Prague, Prague, Czechia; ^4^Department of Botany and Zoology, Faculty of Science, Masaryk University, Brno, Czechia; ^5^Institute of Parasitology, Biology Centre CAS, České Budějovice, Czechia

**Keywords:** real-time PCR, *Hepatozoon*, *Cytauxzoon*, cat, wildcat, exotic felids, vector-borne parasite

## Abstract

Tick-transmitted apicomplexans of the genera *Cytauxzoon* and *Hepatozoon* affect a wide range of felids worldwide, but little is known about them. Recently, several studies addressed the species circulating in Europe, their distribution, and their hosts. Molecular assays are the method of choice for their detection. Unfortunately, conventional PCRs already described are time- and cost-consuming and specific for either *Hepatozoon* or *Cytauxzoon* detection. This study was developed to evaluate (i) the occurrence of *Cytauxzoon* and *Hepatozoon* in felids using a fast and cost-saving real-time PCR capable of detecting both protozoa simultaneously, (ii) the distribution of *Cytauxzoon* and *Hepatozoon* species in north-eastern Italy, and (iii) the involvement of other susceptible felid hosts in the same area. An SYBR^®^ Green-based real-time PCR with primers targeting the 18S-rRNA was validated and applied to 237 felid samples, i.e., whole blood from 206 domestic cats and 12 captive exotic felids, and tissues from 19 wildcats. Positive results were obtained by melting temperature curve analysis due to the specific melting peak (i.e., 81°C *Cytauxzoon* spp.; 78–78.5°C *Hepatozoon* spp.). Positive samples were subjected to conventional PCR, followed by sequencing for species identification. Phylogenetic analyses were performed to assess relatedness among European isolates. Data on domestic cats (age class, sex, origin, management, and lifestyle) were recorded, and statistical analyses were performed to identify potential risk factors. A total of 31 (15%) domestic cats were positive for *Hepatozoon* spp. (i.e., 12 for *H. felis,* 19 for *H. silvestris*), while six (2.9%) for *C. europaeus*. The prevalence of *Hepatozoon felis* was significantly (*p* < 0.05) higher in domestic cats, while *H. silvestris* was higher in strays and animals from the Eastern region (i.e., Friuli-Venezia Giulia). *Cytauxzoon europaeus* was detected only in stray cats from Friuli-Venezia Giulia (province of Trieste). Among captive felids, one tiger was infected with *H. felis* and another with *H. silvestris*; eight out of 19 (42%) wildcats were positive for *Hepatozoon* spp. (i.e., six with *H. felis*, two with *H. silvestris*) and four out of 19 (21%) for *Cytauxzoon europaeus*. Outdoor lifestyle and origin (i.e., Friuli-Venezia Giulia region) were the most relevant risk factors for *H. silvestris* and *C. europeus* infections. Conversely, *H. felis* was most frequently isolated from domestic cats, suggesting different modes of transmission.

## 1. Introduction

*Cytauxzoon* spp. and *Hepatozoon* spp. are the etiologic agents of cytauxzoonosis in felids and hepatozoonosis in a wide range of animals worldwide. The first description of the genus *Cytauxzoon* was recorded in a domestic cat in the USA and was named *Cytauxzoon felis* (1). A different species of *Cytauxzoon* was molecularly recorded in Europe [Spain (2, 3), France (4–6), Portugal (7), Switzerland (6, 8), Germany (9, 10), Luxembourg (10), Romania (10), Czechia (10), Russia (11), and Italy (12–16)] in wild and domestic felids and named *Cytauxzoon* sp. until 2020.

In fact, three species affecting wild and domestic European felids have recently been described and defined as *Cytauxzoon europaeus, Cytauxzoon banethi*, and *Cytauxzoon otrantorum* (10).

Different clinical presentations have been associated with the American and European *Cytauxzoon* species. Although data are still lacking, *Cytauxzoon* in Europe seems to be less virulent than the *C. felis* described in domestic cats in the USA. In fact, no clinical findings are commonly detectable in cats infected with the European *Cytauxzoon* species, which usually present with subclinical infections and are rarely associated with mild anemia (17). However, erythroparasitemia may persist in supposedly healthy cats, which may serve as a reservoir for European *Cytauxzoon* species (12). On the other hand, *C. felis* infections in domestic cats present with generalized rather than specific clinical manifestations, including fever, lethargy, anorexia, dehydration, icterus, pallor of the mucous membrane, dyspnea, and progressively worsening anemia; most infected cats die within 1 week of the onset of clinical signs (18, 19).

*Hepatozoon* spp. has been described in several host species, such as mammals, reptiles, birds, and amphibians (20). In particular, three *Hepatozoon* species have been described and reported in wild and domestic felids in Europe: *H. felis*, *H. silvestris*, and *H. canis. Hepatozoon* infections are widespread and have been reported in Spain (3), France (4, 21), Portugal (22), Cyprus (23), Germany (24), Austria (25), Greece (26), and Italy (15, 27, 28).

*Hepatozoon felis* infection is often subclinical and detected accidentally by observation of stained blood smears. When present, clinical manifestations are not specific and include anemia, elevated creatine kinase, and lactate dehydrogenase values (29). Clinical manifestations of *H. silvestris* in case reports have been under-reported in the scientific literature. Kegler et al. (24) reported a cat in Switzerland infected with *H. silvestris* with fatal lymphoplasmacytic and histiocytic myocarditis, weakness, and anorexia.

Finally, *H. canis* has also been described in dogs and cats, although clinical signs in cats are occasionally reported (3, 27, 30).

The observation of specific parasitic inclusions (i.e., merozoites) in red blood cells suggests piroplasmic infection and cytauxzoonosis. Unfortunately, the stained blood smear is not sufficient to make a diagnosis, and molecular analysis is strongly recommended. Moreover, since cytauxzoonosis is characterized by a low burden of merozoites circulating in the bloodstream in both acute and chronic phases of the infection, molecular analysis represents the gold standard for the diagnosis due to its high sensitivity and ability to identify the *Cytauxzoon* species involved (31).

Although the detection of *Hepatozoon* gamonts may be sufficient for a hepatozoonosis diagnosis, molecular detection is strongly suggested because hepatozoonosis in felids is usually asymptomatic and characterized by low parasitemia in about 1% of infected white blood cells (32).

The literature has described conventional polymerase chain reaction (cPCR) assays for *Cytauxzoon* and *Hepatozoon* detection, usually targeting 18S rRNA, a highly conserved gene for both protozoa (8, 23, 33–38), and cytochrome B and cytochrome C oxidase subunit I (COI), mitochondrial protein-coding genes, which are more specific for *Cytauxzoon* species identification (10). Real-time polymerase chain reaction (real-time PCR) may be a reasonable alternative for the rapid screening of large numbers of samples. This method is time-saving because the electrophoresis gel analysis is not required, and the fluorescence analysis allows the operator to collect data while real-time PCR is running. In 2007, Criado-Fornelio et al. (39) developed a real-time PCR assay for the detection of *Hepatozoon* spp. in canine and feline blood samples, and the procedure appeared to be more sensitive than conventional PCR in feline samples. Recently, real-time PCR assays targeting the piroplasmid 18S-rRNA gene have been used to individually detect *Cytauxzoon* spp. and *Hepatozoon* spp. in tissue samples and blood from domestic and wild felids in Europe (6, 16). However, to our knowledge, no assay has been developed to simultaneously detect *Cytauxzoon* spp. and *Hepatozoon* spp. DNA.

In light of the above considerations, this study aimed to (i) evaluate the occurrence of *Hepatozoon* spp. and *Cytauxzoon* in felids using real-time PCR for the simultaneous detection of both pathogens in north-eastern Italy, (ii) assess the distribution of *Cytauxzoon* and *Hepatozoon* species and their genetic diversity in felids, and (iii) study the involvement of felines other than domestic cats.

## 2. Materials and methods

### 2.1. Study area

The molecular survey was developed in north-eastern Italy, where *Cytauxzoon* and *Hepatozoon* circulation in domestic cats has already been proven (12, 15). In particular, the regions of Veneto (Site 1), Friuli-Venezia Giulia (Site 2), and Trentino-Alto Adige (Site 3) were investigated. Blood samples collected from captive felids living in zoological parks in other regions (i.e., Lazio, Piedmont) were also included.

### 2.2. Felid sampling and individual data collection

Different species of felids were sampled: domestic cats (*Felis silvestris catus*) from Sites 1, 2, and 3, European wildcats (*Felis silvestris silvestris*) from Site 2, and captive exotic felids, i.e., tigers (*Panthera tigris*), leopards (*Panthera pardus*), lions (*Panthera leo*), and caracals (*Caracal caracal*), living in zoological parks located in Site 1, except for two animals from zoological parks of the Lazio and Piedmont regions. Whole blood was collected from domestic cats and captive exotic felids during routine clinical visits and/or surgical procedures (not dependent on this research study) thanks to the collaboration of some private veterinary practices/clinics operating in the study area. Tissues (i.e., heart, lung, spleen, liver, lymph nodes, and blood clots) were collected from European wildcats found dead in the monitored areas and stored at −20°C until post-mortem examination. Individual data were recorded for each animal sampled. In particular, origin (i.e., Site 1, Site 2, Site 3), sex, age classes (i.e., <12 months, from 12 to 36 months, >36 months), management (i.e., domestic, stray), and lifestyle (i.e., indoor, outdoor) were recorded for domestic cats; origin, sex, and age classes (i.e., <12 months = sub-adults; ≥12 months = adults) were recorded for captive exotic felids and European wildcats.

### 2.3. DNA extraction, molecular analysis, and sequencing

DNA was extracted from 200 μL of whole blood or 25 mg of organs/clots using the NucleoSpin^®^ Tissue kit (Macherey-Nagel, Düren, Germany) according to the manufacturer’s instructions.

Real-time PCR was used to analyze the DNA extracts with the QuantiNova SYBR^®^ Green PCR Kit (QIAGEN Group, Hilden, DE) and primers targeting a 373 bp fragment of the piroplasmid 18S-rRNA gene (Table 1).

**Table 1:** Primers used for molecular detection of *Hepatozoon* spp. and *Cytauxzoon* spp.

Gene	Primer	Sequence	Amplicon size (bp)	References
18S-rRNA	PIROPLASMID-F	CCAGCAGCCGCGGTAATTC	373	(40)
	PIROPLASMID-R	CTTTCGCAGTAGTTYGTCTTTAACAAATCT		
cytochrome B	Cytaux_cytb_F1	CTTAACCCAACTCACGTACC	1,434	(41)
	Cytaux_cytb_R3	GGTTAATCTTTCCTATTCCTTACG		
	Cytaux_cytb_Finn	ACCTACTAAACCTTATTCAAGCRTT	1,333	(10)
	Cytaux_cytb_Rinn	AGACTCTTAGATGYAAACTTCCC		

The assay was performed using the Roche LightCycler^®^ 96 thermocycler (Roche, Basel, Switzerland) with the following amplification cycle: incubation at 95°C for 2 min, followed by 45 cycles of amplification steps at 95°C for 5 s and 60°C for 10 s, final at 95°C for 10 s, 65°C for 1 min, and 97°C for 1 s. Melting curve analysis was performed by continuously monitoring fluorescence as the temperature decreased from 95°C to 65°C. Positive (i.e., DNA from sequenced field samples) and negative (no DNA added) controls were added in each PCR reaction.

**Figure 1:**
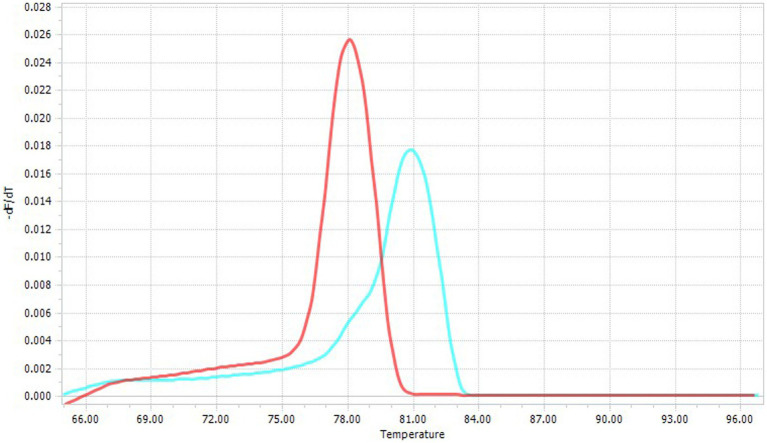
Specific melting temperatures of *Cytauxzoon* spp. (T_m_ = 81°C; blue line) and *Hepatozoon* spp. (T_m_ = 78–78.5°C, red line).

Fluorescence specificity and genus identification were achieved by melting temperature (T_m_) curve analysis (i.e., *Cytauxzoon* spp. *T*_m_ = 81°C; *Hepatozoon* spp. *T*_m_ = 78–78.5°C) (Figure 1) (42). Amplicons from *Hepatozoon* spp. positive samples were submitted directly for sequencing, as the 18S-rRNA gene is sufficient to identify the *Hepatozoon* species. Conversely, *Cytauxzoon* spp. positive samples were processed by a nested PCR targeting the cytochrome B gene using primers (Table 1) and protocols already described (10), considering that a more variable gene is required to identify *Cytauxzoon* species.

Amplicons from real-time PCR and nested PCR were Sanger sequenced (Macrogen Spain, Madrid, Spain) and the obtained nucleotide sequences were compared to those deposited in GenBank^®^ using BLAST software^1^ (accessed: 15 October 2022).

### 2.4. Phylogenetic analysis

The obtained sequences were subjected to BLAST^®^ analysis, and a collection of closely related *H. felis* and *H. silvestris* sequences was identified and downloaded from Genbank^®^. Moreover, a representative sample of *H. canis* sequences was also downloaded to be used as an outgroup in the phylogenetic analysis. Selected sequences were aligned with the ones obtained in the present study using MAFFT (43). A neighbor-joining tree was reconstructed using MEGA X (44), selecting as the substitution model the one with the lowest Akaike information criterion (AIC), calculated with JModeltest (45). The reliability of inferred clades was inferred by performing 1000 bootstrap replicates.

A comparable approach was applied to the analysis of *C. europaeus* sequences. The obtained sequences were subjected to BLAST^®^ analysis, and a collection of closely related *C. europaeus* sequences was also identified and downloaded from Genbank^®^. Alignment and phylogenetic analysis were then performed as previously described for Hepatozoon. *Cytauxzoon felis* sequences were also downloaded to be used as an outgroup.

### 2.5. Data analysis

Differences in infection rates among domestic cat populations with respect to individual factors were statistically evaluated through Pearson’s chi-squared test or Fisher’s exact test, as appropriate, using R software 4.1.2. The considered factors were sex (i.e., male, female), age class (i.e., <12 months, from 12 to 36 months, >36 months), origin (i.e., Site 1, Site 2, Site 3), lifestyle (i.e., indoor, outdoor), and management (i.e., domestic, stray). Captive felids and wildcats were not included in the statistical analysis due to the small number of samples.

## 3. Results

### 3.1. Description of felid populations

A total of 237 felid samples were collected and analyzed by real-time PCR: 206 domestic cats, 12 captive exotic felids, and 19 wildcats.

Of the domestic cat population, 70.9% (*n* = 146/206) were from Site 1, followed by 19.4% (*n* = 40/206) from Site 2, and 9.7% (*n* = 20/206) from Site 3. Most of them were domestic cats (*n* = 131, 63.6%), and the rest were strays from street colonies (*n* = 75, 36.4%). Domestic cats had mostly an outdoor lifestyle (*n* = 139, 67.5%) and were evenly distributed among sex and age classes. Individual data for the sampled domestic cats are shown in Table 2.

**Table 2:** Description of individual data of the domestic cat population distributed among the three investigated sites.

		Site 1 n (%)	Site 2 n (%)	Site 3 n (%)	Total n (%)
Sex	M	74 (50.7)	14 (35.0)	12 (60.0)	100 (48.5)
	F	70 (47.9)	26 (65.0)	8 (40.0)	104 (50.5)
	NR^1^	2 (1.4)	0 (0.0)	0 (0.0)	2 (1.0)
Age class (months)	<12	51 (34.9)	9 (22.5)	5 (25.0)	65 (31.6)
	12–36	41 (28.1)	17 (42.5)	8 (40.0)	66 (3 2.0)
	>36	43 (29.5)	12 (30)	7 (35.0)	62 (30.1)
	NR^1^	11 (7.5)	2 (5.0)	0 (0.0)	13 (6.3)
Management	Domestic	91 (62.3)	20 (50.0)	20 (100.0)	131 (63.6)
	Stray	55 (37.7)	20 (50.0)	0 (0.0)	75 (36.4)
Lifestyle	Indoor	42 (28.8)	18 (45.0)	7 (35.0)	67 (32.5)
	Outdoor	104 (71.2)	22 (55.0)	13 (65.0)	139 (67.5)
Total		146 (70.9)	40 (19.4)	20 (9.7)	206 (100.0)

1 Not reported.

The 12 captive exotic felids included four tigers (*Panthera tigris*), two lions (*Panthera leo*), three leopards (*Panthera pardus*), and one caracal (*Caracal caracal*) from different zoological parks located in Sites 1 and 2 tigers came from zoological parks in the regions of Lazio and Piedmont.

The wildcats that were road-killed or found dead in Site 2 were 10 (52.6%) males and 9 (47.4%) females, and their age was estimated by dental evaluation, classifying them as adults (*n* = 17/19, 89.5%) or sub-adults (*n* = 2/19, 10.5%).

### 3.2. Analysis results

Real-time PCR detected *Hepatozoon* spp. infection in 41/237 (17.3%) felids and *Cytauxzoon* spp. in 10/237 (4.2%). A total of 31/206 (15%) domestic cats were positive for *Hepatozoon* spp. (i.e., 12 *H. felis*, 19 *H. silvestris*) and 6/206 (2.9%) for *C. europaeus*. Among captive felids, one tiger was infected with *H. felis* and another one with *H. silvestris*; 8/19 (42%) wildcats were positive for *Hepatozoon* spp. (i.e., six *H. felis*, two *H. silvestris*) and 4/19 (21%) for *Cytauxzoon europaeus*. No co-infections were detected. The detailed results are summarized in Table 3.

**Table 3:** Distribution of positive cases by felid species, melting temperatures, and sequencing.

Felids	n/tot (%)	T_m_ (°C)	Real-time PCR	n/tot	Sequencing	% Identity
Domestic cats	31/206 (15)	78/78.5	*Hepatozoon* spp.	12/31 19/31	*H. felis* *H. silvestris*	99.7–100% 97–100%
	6/206 (2.9)	81	*Cytauxzoon* spp.	6/6	*C. europaeus*	100%
Wildcats	8/19 (42.1)	78/78.5	*Hepatozoon* spp.	6/8 2/8	*H. felis* *H. silvestris*	97.3–99.7% 99.7%
	4/19 (21)	81	*Cytauxzoon* spp.	4/4	*C. europaeus*	98.8–100%
Exotic felids	2/12 (16.7)	78/78.5	*Hepatozoon* spp.	1/2 1/2	*H. felis* *H. silvestris*	99.7% 99.7%

*Cytauxzoon*-positive cats were all strays from the province of Trieste in Site 2, whereas *Hepatozoon* spp.-positive cats were distributed in all investigated sites. Individual data concerning infected domestic cats are reported in Table 4.

**Table 4:** Distribution of positive cases by individual factors in the cat population.

	Variables	Tested	*C. europaeus* n (%)		*H. felis* n (%)		*H. silvestris* n (%)	
Sex	M	100	1 (1.0)		5 (5.0)		10 (10.0)	
	F	104	5 (4.8)		7 (6.7)		9 (8.6)	
	NR^1^	2	0 (0.0)		0 (0.0)		0 (0.0)	
Age class (months)	<12	65	0 (0.0)		5 (7.7)	*	5 (7.7)	
	12–36	66	2 (3.0)		0 (0.0)		8 (12.1)	
	>36	62	3 (4.8)		6 (9.7)		5 (8.1)	
	NR^1^	13	1 (7.7)		1 (7.7)		1 (7.7)	
Region	Site 1	146	0 (0.0)	*	7 (4.8)		10 (6.8)	*
	Site 2	40	6 (15.0)		2 (5.0)		9 (22.5)	
	Site 3	20	0 (0.0)		3 (15.0)		0 (0.0)	
Management	Domestic	131	0 (0.0)	*	11 (8.4)	*	6 (4.6)	*
	Stray	75	6 (8.0)		1 (1.3)		13 (17.3)	
Lifestyle	Indoor	67	0 (0.0)		4 (6.0)		3 (4.5)	
	Outdoor	139	6 (4.3)		8 (5.8)		16 (11.5)	
Total		206	6 (2.9)		12 (5.8)		19 (9.2)	

1 Not reported.

* Significant differences (*p* < 0.05) based on Pearson’s chi-squared test or Fisher’s exact test.

**Figure 2:**
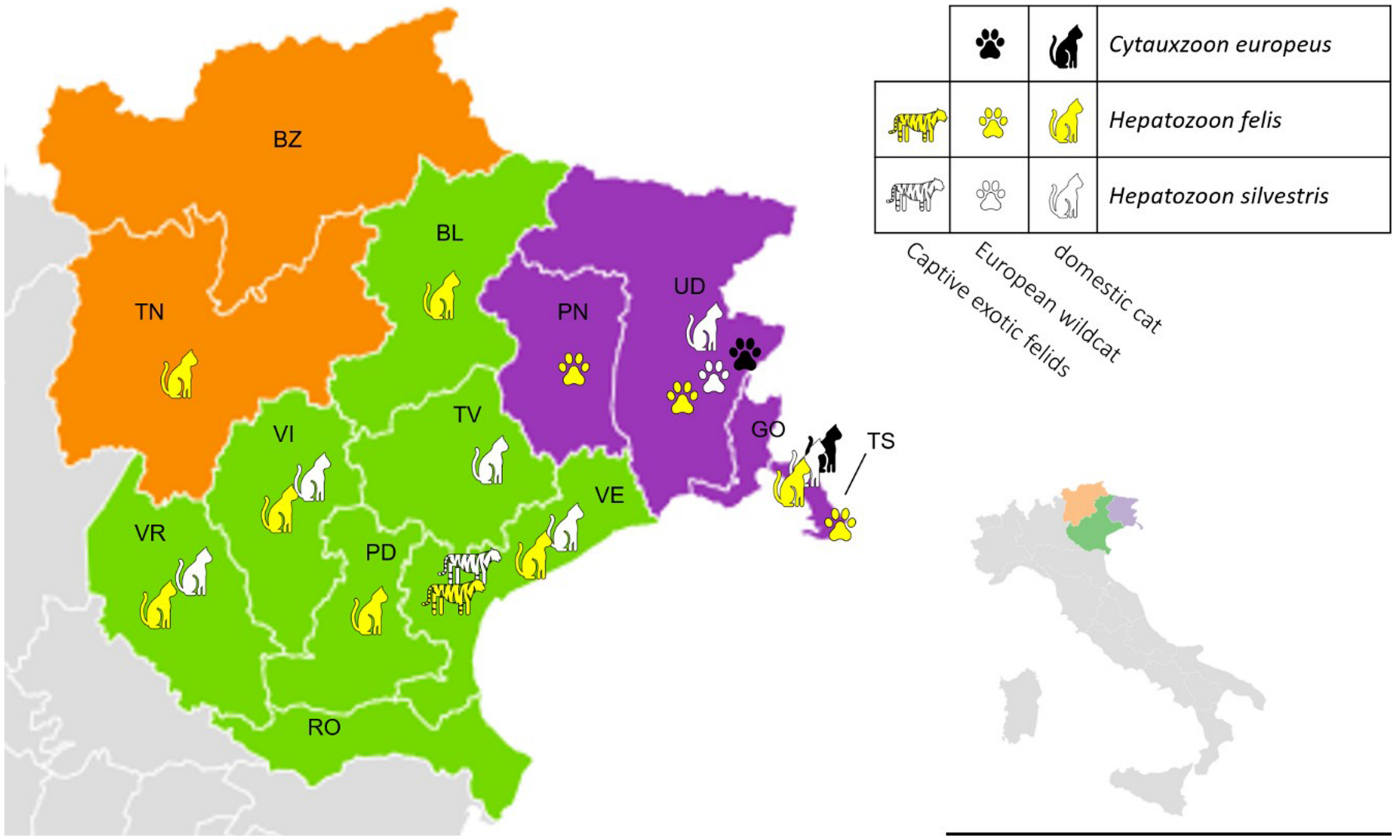
Distribution of *Cytauxzoon europeus*, *Hepatozoon felis*, and *Hepatozoon silvestris* among different felid hosts in north-eastern Italy by regions and provinces (green -Veneto region – Site 1: BL, Belluno; PD, Padova; RO, Rovigo; TV, Treviso; VE, Venezia; VI, Vicenza; VR, Verona; purple – Friuli-Venezia Giulia region – Site2: GO, Gorizia; PN, Pordenone; TS, Trieste; UD, Udine; orange – Trentino-Alto Adige region – Site 3: BZ, Bolzano; TN, Trento).

A significantly higher prevalence (*p* < 0.05) of *C. europeus* and *H. silvestris* infections was found in stray cats and animals living in Site 2. On the other hand, domestic cats were statistically more often infected by *H. felis* than stray cats, as were kittens younger than 1 year or adults older than 3 years (Table 4). Among captive felids, only two tigers were positive for *Hepatozoon* spp., one for *H. felis* and one for *H. silvestris*. Both were from a zoological park in the Veneto region (Site 1). No exotic felid species tested positive for *Cytauxzoon* spp. All positive wildcats were adults (older than 1 year), mostly females, from several provinces of Site 2 (i.e., Trieste, Udine, and Pordenone). The distribution of *C. europeus*, *H. felis*, and *H. silvestris* in domestic cats, European wildcats, and captive exotic felids is shown in Figure 2.

All sequences isolated from domestic cats, wildcats, and tigers of *C. europaeus* (from OP757647 to OP757655), *H. felis* (from MZ227585 to MZ227594 and from OP693639 to OP693646), and *H. silvestris* (from MZ227596 to MZ22611 and from OP694164 to OP694169) have been deposited in GenBank^®^.

### 3.3. Phylogenetic analysis

**Figure 3:**
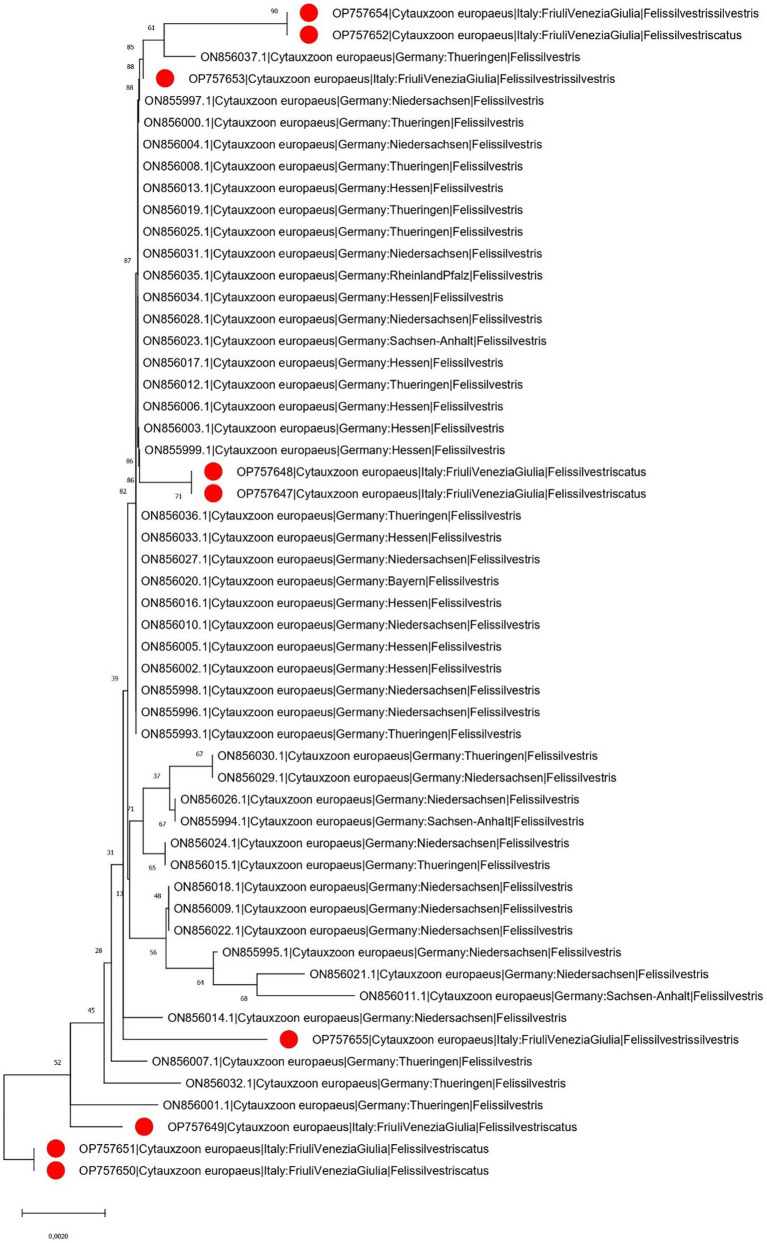
Neighbor-joining phylogenetic tree reconstructed based on a region of the cytochrome B gene (870 bp) of *C. europaeus* using the HKY + G substitution model. Bootstrap support is reported near the corresponding node. The isolates whose sequences have been obtained in the present study are marked with a red dot in the phylogenetic tree near to the respective sequence name. For graphical reasons, the *C. felis* outgroup is not shown. The country of collection and host were annotated in the sequence name when available.

**Figure 4:**
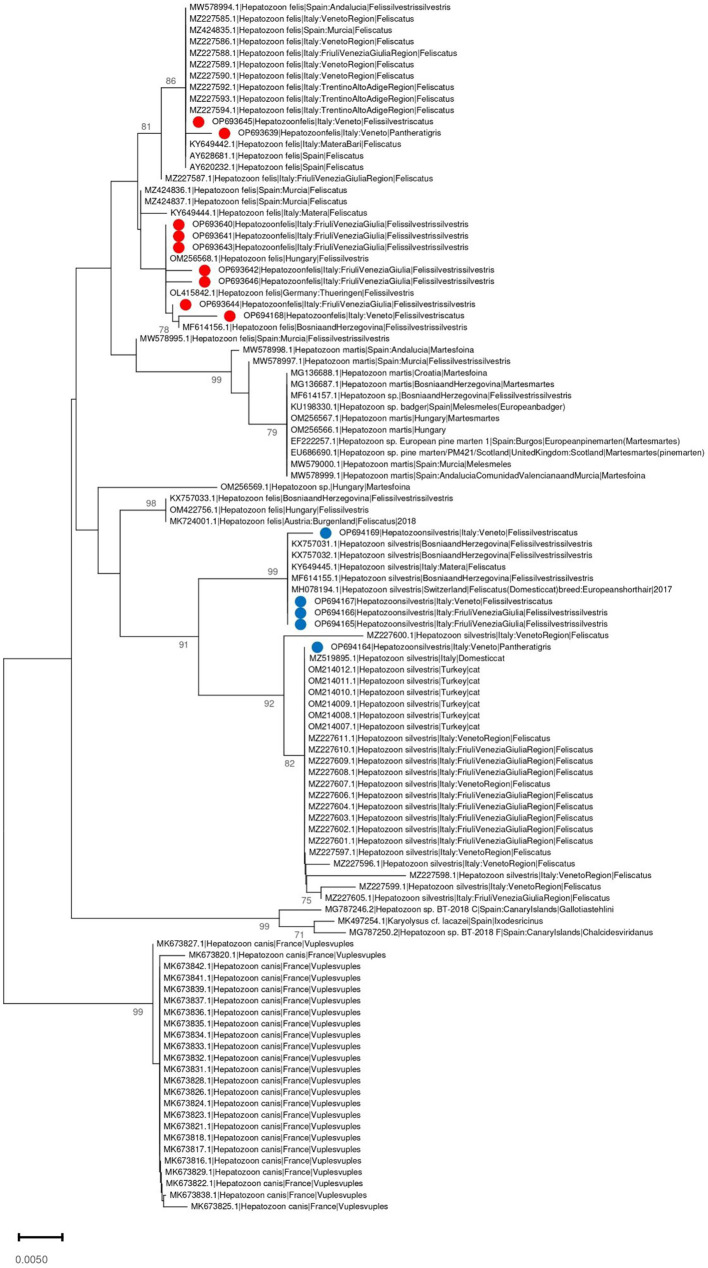
Neighbor-joining phylogenetic tree reconstructed based on a region of the 18S-rRNA gene (350 bp) of *Hepatozoon* spp. using the T92 + G substitution model. Bootstrap support is reported near the corresponding node. *H. felis* and *H. silvestris* isolates whose sequences have been obtained in the present study are marked respectively with a red or blue dot in the phylogenetic tree near to the corresponding sequence name. The country of collection and host were annotated in the sequence name when available.

The phylogenetic tree of *Cytauxzoon europaeus* revealed that the obtained sequences were different and sparse along the tree, whereas *Hepatozoon* species formed well-separated clades. The *Hepatozoon felis* sequences obtained in the present study were part of two clusters that also included, besides other Italian strains, sequences from Spain, Hungary, and Germany. Similarly, *H. silvestris* strains were part of two clades, one that included sequences from Italy, Switzerland, and Bosnia and Herzegovina, and the other comprising Italian and Turkish strains (Figures 3, 4).

## 4. Discussion

Vector-borne diseases have stimulated the interest of the scientific community in the last decades, and indeed, the epidemiologic data for *Cytauxzoon* spp. and *Hepatozoon* spp. in wild and domestic felids are continuously updated (6, 10, 46). In Italy, the circulation of *Cytauxzoon* spp. and *Hepatozoon* spp. has been reported in both domestic and wildcats (12, 15, 27, 47), highlighting the need to better investigate their circulating species and genotypes. A recent survey of *Cytauxzoon* spp. and *Hepatozoon* spp. infections in asymptomatic domestic cats confirmed the presence and establishment of a domestic cycle for both protozoa in northeastern Italy. Hepatozoonosis was fairly evenly distributed in all the investigated regions, whereas cytauxzoonosis was strictly limited to the province of Trieste in the Friuli-Venezia Giulia region (15). The present study (i) updates the epidemiological data on domestic cats in those same areas using a new, fast, and sensitive molecular procedure (real-time PCR) able to detect and differentiate simultaneously both protozoa, (ii) adds information on the protozoan species and their distribution in north-eastern Italy, and (iii) investigates other potential felid hosts in the same areas and their potential role in the transmission of both protozoa.

Out of 237 animals, 4.2% were infected with *Cytauxzoon*. In particular, the 10 positive animals were six stray cats and four wildcats coming from the same site (Site 2 – Friuli-Venezia Giulia region), where the first Italian report of *Cytauxzoon* was identified in 2012 (12) and recognized as endemic about 10 years later (14). The province of Trieste is close to Slovenia and is considered an ecological corridor for wildlife movements (48). In addition, the province is characterized by a large peri-urban area overlapping with a sylvatic environment inhabited by wildcats, suggesting the possible role of these wild felids as reservoirs. Moreover, the Eurasian lynx is present in the region (49, 50) and may be involved in parasite circulation, as previously reported (51).

In fact, the stray cats at Site 2, including mostly street colony cats, were statistically more infected than domestic cats, and this supports the hypothesis that animals with an exclusively outdoor lifestyle, living virtually in sympatry with sylvatic species and without regular controls and treatments, are more exposed to the risk of infection. No captive exotic felid was found to be infected with *Cytauxzoon* spp. Considering that all tested animals lived in zoological parks in Site 1 (Veneto region), this could be related to the absence of cytauxzoonosis in domestic or wildcats in the considered area. On the other hand, exotic felids are susceptible hosts for other *Cytauxzoon* species such as *C. felis* and *C. manul* (18).

In north-eastern Italy, *Cytauxzoon* spp. was first reported in domestic cats in 2012 (12) and in wildcats in 2016 (47); however, species identification was not achieved until recently, when a new molecular approach targeting more variable genes (i.e., cytochrome B and cytochrome C oxidase subunit I – COI) allowed phylogenetic analyses (10). In this study, all sequences had a percentage identity of 99–100% with reference sequences of *C. europeus*, the most common species isolated in felids in Europe (10).

The obtained *Cytauxzoon* phylogenetic analysis showed a scenario that partially contrasts with what was observed in Germany, where most of the sequences were identical, with few exceptions. Unfortunately, the limited sequence availability and the lack of proper knowledge about the epidemiology of this parasite prevent any definitive conclusion, and only speculative hypotheses can be advocated. Therefore, more extensive and systematic studies should be performed to formally evaluate the prevalence of infection and to investigate the spreading patterns and the involved countries.

The presence of wildcats in the Friuli-Venezia Giulia region is known, also thanks to the possible movement of these animals across the Alps from nearby Slovenia (48). Recently, the distribution of European wildcats in Italy has been updated (52), and the data collected shows that wildcats are expanding their territory toward the northern part of the Veneto region and the southern areas of the Trentino-Alto Adige region (53, 54).

Out of 237 animals, 17.3% were infected with *Hepatozoon* spp. Specifically, 41 positive animals were recorded (i.e., 31 domestic cats, eight wildcats, and two tigers).

A significantly higher prevalence (*p* < 0.05) of *H. silvestris* infection was observed in stray cats and animals living in Site 2, suggesting that a free-roaming lifestyle increases exposure to vectors. *H. silvestris* was also isolated in domestic cats in Site 1, most of them coming from the province of Verona, followed by the provinces of Vicenza, Treviso, and Belluno. These positive results are particularly interesting because they seem to follow the recent movements of wildcats in the Italian territory. Even if the infected cats of Site 1 were evenly divided between strays and domestic animals, all of them had an outdoor lifestyle. The main risk of exposure is thus related to living outdoors and coming into contact with ticks, although it must be considered that other modes of transmission could be possible. In the *H. canis* and *H. felis* life cycles, vertical transmission from mother to offspring during pregnancy has been proven (30, 32), and in the *H. americanum* life cycle, predation on infected prey has been demonstrated to be a mode of transmission (32). In felids, no information is available, and it can only be assumed that vertical and/or horizontal transmission by ingestion of infected prey may be possible. These considerations justify the *H. felis* results. First, *H. felis* is fairly evenly distributed in all areas investigated and affects both domestic and wild felids. In particular, *H. felis* was found more frequently in domestic cats and animals younger than 1 year and older than three, with statistically significant prevalence values. If outdoor habits were a significant risk factor for domestic cat infection with *C. europeus* and *H. silvestris*, they do not seem to be significant for *H. felis*; in fact, positive cats were evenly distributed between indoor and outdoor animals, some of which were infected in areas where wild felids are not yet reported (e.g., the southern areas of Site 1), suggesting a domestic cycle of *H. felis*. In addition, younger animals were more likely to be infected by the vertical route (30) along with older animals because they had more time to be exposed to the infection through the ingestion of infected arthropods and/or infected prey. The same considerations could justify the isolation of *H. felis* from wildcats; indeed, all the animals were adults and came from different provinces of Site 2 (i.e., the Trieste, Udine, and Pordenone provinces).

None of the 12-36-month-old cats tested positive for *H. felis.* Since hepatozoonosis is still under-investigated and no data are available on pathogenesis and host immune response, it could be assumed that (i) a young cat with a competent immune system is likely to be able to face and overcome the infection, (ii) no immunological memory is formed after the infection, and (iii) repeated exposure throughout life leads cats to new infections as they become older.

Among the captive wild felids, two tigers with hepatozoonosis came from the same zoological park in the province of Venezia in Site 1, where both *H. felis* and *H. silvestris* were isolated in domestic cats. To our knowledge, this is the first description of *H. felis* and *H. silvestris* in captive tigers in Italy, as three tigers from a zoological park located in southern Italy were found positive for *H. canis* (55).

The *H. felis* sequence analysis revealed two distinct clusters, whose features support the above-mentioned scenario. One contained sequences mainly from domestic cats, with only three exceptions (i.e., the tiger and two wildcats, one from Italy and one from Spain), while the other comprised only strains from wildcats, originating from Italy and Eastern Europe, with the sole exception of one strain detected from a wildcat in the present study. Therefore, despite the separation between the domestic and wild cycles, it is likely that some strain exchange occurs in both directions. Finally, the relationship between sequences obtained from captive and domestic animals supports the involvement of a domestic cycle, although the precise contact pathway remains unclear. Similar evidence emerged from *H. silvestris*, where a close relationship was found between Italian strains obtained from *Panthera tigris* and a domestic cat. Also in this case, two clusters were observed, one containing sequences mainly, but not only, from wildcats, linked to strains collected in Eastern European countries, and another group containing only strains from domestic cats (the tiger being the only exception). In the latter, the clustering with Turkish strains can hardly be explained, supporting the speculative hypothesis about the role of human and pet travel in parasite dispersal.

The observation of merozoites in red blood cells and *Hepatozoon* gamonts in white blood cells in stained feline blood smears is strongly suggestive of these protozoan infections. However, because cytauxzoonosis and hepatozoonosis usually present a low parasite burden in feline hosts (8, 15, 32), the blood smear is not a sensitive method, and molecular procedures may be considered the method of choice for the diagnosis. Several studies support the higher sensitivity of molecular procedures compared to stained blood smears in the diagnosis of hepatozoonosis. In 2006, a study reported that 32% of cats were positive for *Hepatozoon* spp. by PCR, while only 0.7% had gamonts in blood smears (56). Similarly, Pereira et al. (57) described that 12.5% of cats in Cape Verde were PCR-positive for *H. felis*, with no gamonts observed in blood smears. Conventional PCR is currently the most common method for the individual detection of *Hepatozoon* spp. and *Cytauxzoon* spp.; however, it has some limitations (e.g., time and cost) that can be overcome by alternative solutions, such as a real-time PCR protocol.

The real-time PCR procedure adopted in this study was designed to guarantee a high-sensitivity protocol that can (i) simultaneously detect and (ii) differentiate both *Cytauxzoon* spp. and *Hepatozoon* spp. DNA by rapid melting curve analysis, (iii) be applied to different matrices (from blood to different tissues and organs), and (iv) quickly screen a consistent number of samples. Because of the low sensitivity of blood smear observations, the lack of serological tests on the market, and the limitation of the molecular procedures described in the literature to detect only one pathogen at a time, this protocol represents a good choice for simultaneously diagnosing hepatozoonosis and cytauxzoonosis.

Conventional PCR was successively adopted to further characterize the involved species and strains in positive samples by Sanger sequencing, allowing alignment and phylogenetic analysis. In conclusion, this study updated the epidemiologic data on cytauxzoonosis and hepatozoonosis in feline hosts through a new, fast, and sensitive real-time PCR, providing new information on the species and strains involved, their genetic correlation with European isolates, susceptible feline hosts, and their distribution in north-eastern Italy.

## Data availability statement

The original contributions presented in the study are included in the article/supplementary material, further inquiries can be directed to the corresponding author.

## Ethics statement

Ethical review and approval was not required for the animal study because blood collection on felids was performed during routine veterinary procedures not depending on this research project. Written informed consent was obtained from the owners for the participation of their animals in this study.

## Author contributions

MG, GS, and AFdR: conceptualization. MG, GD, CT, and GF: methodology. GD and CT: validation. MG, GS, EM, and PB: investigation. GF: software. GS: resources and project administration. MG: data curation and writing—original draft preparation. GS, AFdR, and DM: writing—review and editing. AFdR and GS: supervision. All authors read and agreed to the published version of the manuscript.

## Funding

This research was supported by the Department of Animal Medicine, Production, and Health of the University of Padua (BIRD193835, 2019).

## Conflict of interest

The authors declare that the research was conducted in the absence of any commercial or financial relationships that could be construed as a potential conflict of interest.

## Publisher’s note

All claims expressed in this article are solely those of the authors and do not necessarily represent those of their affiliated organizations, or those of the publisher, the editors and the reviewers. Any product that may be evaluated in this article, or claim that may be made by its manufacturer, is not guaranteed or endorsed by the publisher.

## Footnotes

^1^https://blast.ncbi.nlm.nih.gov/Blast
